# Coptidis Rhizoma Prevents Heat Stress-Induced Brain Damage and Cognitive Impairment in Mice

**DOI:** 10.3390/nu9101057

**Published:** 2017-09-23

**Authors:** Minho Moon, Eugene Huh, Wonil Lee, Eun Ji Song, Deok-Sang Hwang, Tae Hee Lee, Myung Sook Oh

**Affiliations:** 1 Department of Biochemistry, College of Medicine, Konyang University, 158, Gwanjeodong-ro, Seo-gu, Daejeon 35365, Korea; hominmoon@konyang.ac.kr (M.M.); thddmswl1028@gmail.com (E.J.S.); 2 Department of Life and Nanopharmaceutical Sciences, Graduate School, Kyung Hee University, 26 Kyungheedae-ro, Dongdaemun-gu, Seoul 02447, Korea; eugenehuh@khu.ac.kr (E.H.); rocknrollbb@gmail.com (W.L.); 3 Department of Herbal Pharmacology, College of Korean Medicine, Graduate School, Kyung Hee University, 26 Kyungheedae-ro, Dongdaemun-gu, Seoul 02447, Korea; 4 Department of Korean Gynecology, College of Korean Medicine, Kyung Hee University, 26 Kyungheedae-ro, Dongdaemun-gu, Seoul 02447, Korea; soulhus@gmail.com; 5 Department of Formulae Pharmacology, School of Oriental Medicine, Gachon University, 1342 Seongnamdae-ro, Sujeong-gu, Seongnam 13120, Korea; ophm5418@gachon.ac.kr; 6 Department of Oriental Pharmaceutical Science, College of Pharmacy, Kyung Hee University, 26 Kyungheedae-ro, Dongdaemun-gu, Seoul 02447, Korea

**Keywords:** Coptidis Rhizoma, antipyretic, heat stress, neuroinflammation, cognitive functions

## Abstract

Heat stress conditions lead to neuroinflammation, neuronal death, and memory loss in animals. Coptidis Rhizoma (CR) exhibits potent fever-reducing effects and has been used as an important traditional medicinal herb for treating fever. However, to date, the effects of antipyretic CR on heat-induced brain damages have not been investigated. In this study, CR significantly reduced the elevation of ear and rectal temperatures after exposure to heat in mice. Additionally, CR attenuated hyperthermia-induced stress responses, such as release of cortisol into the blood, and upregulation of heat shock protein and c-Fos in the hypothalamus and hippocampus of mice. The administration of CR inhibited gliosis and neuronal loss induced by thermal stress in the hippocampal CA3 region. Treatment with CR also reduced the heat stress-induced expression of nuclear factor kappa β, tumor necrosis factor-α, and interleukin-1β (IL-1β) in the hippocampus. Moreover, CR significantly decreased proinflammatory mediators such as IL-9 and IL-13 in the heat-stressed hypothalamus. Furthermore, CR attenuated cognitive dysfunction triggered by thermal stress. These results indicate that CR protects the brain against heat stress-mediated brain damage via amelioration of hyperthermia and neuroinflammation in mice, suggesting that fever-reducing CR can attenuate thermal stress-induced neuropathology.

## 1. Introduction

Heat stress, which is mediated by extreme environmental temperature, is a natural stressor that causes systemic metabolic disorders in animals. Recently, many reports have demonstrated that excessive exposure to heat induces various pathophysiological responses such as hyperthermia, water loss, anorexia, weight loss, changes in glucocorticoid levels, vasopressin elevation, increased heart rate, hypoglycemia, gastric hemorrhage, and spermatogenesis dysfunction [1,2]. Moreover, serious heatstroke might result from extreme thermal stress. The hypothalamic-pituitary-adrenal (HPA) axis, when activated by heat stress, is responsible for inhibitory effects on immune functions [3,4]. In addition to the detrimental effects of heat stress on the peripheral immune system, heat stress stimulates neuroinflammatory responses, including the generation of cytokines, in the brain [5]. Furthermore, previous studies have reported that the brain is very susceptible to high temperature exposure; heat stress can lead to modification of the neuronal circuit, neuronal death, neurological defects, spasms, brain atrophy, and neurogenic deficits [5,6]. Particularly, it has been well known that hyperthermia-induced stress is able to cause memory deficits [7,8]. Taken together, heat exposure might induce stress responses, neuroinflammation, neuronal death, and cognitive dysfunction in animals.

There has been a growing interest in natural compounds having antipyretic actions to treat symptoms induced by extremely high environmental temperatures, such as fever and heat stroke [9,10]. For example, *Panax ginseng* C.A. Mey. (Araliaceae) and *Panax quinquefolius* L. (Araliaceae) regulate body temperature under both hypothermic and hyperthermic conditions, or high and low room temperature environments [11]. Additionally, ginsenoside Rb1 from ginseng was demonstrated to increase the expression of heat shock protein 70 in the rat hippocampus [12]. Moreover, in India, traditional healers of the Kancheepuram district commonly use a number of medicinal plants to treat fever, such as *Wattakaka volubilis* (L.f.) Stapf (Asclepiadaceae), *Leucas aspera* (Willd.) Link (Lamiaceae), *Citrus auantifolia* (Christm.) swingle (Rutaceae), *Clerodendrum inerme* (L.) Gaertn (Verbenaceae), and *Vitex negundo* L. (Verbenaceae) [13]. Among a large number of medicinal plants, Coptidis Rhizoma (CR) is considered to be a very effective traditional medicinal herb with potent antipyretic activities. According to traditional literature, CR is a major component of many traditional medicinal prescriptions used to lower fever, soothe coughs, relax blood vessels, and prevent mycoses [14,15,16,17]. In particular, many previous studies clearly demonstrated the fever-reducing effects of CR [18,19]. Berberine, a major bioactive constituent of CR, showed potent thermoregulating activities under both hot and cold conditions, suggesting its antagonizing effects on temperature changes [20]. Moreover, the antifebrile effect of CR may be mediated by regulating the expression of transient receptor potential vanilloid 1 and melastatin 8, in the paraventricular nucleus and supraoptic nucleus of the hypothalamus and dorsal root ganglia [19,21]. Additionally, numerous studies have demonstrated the anti-inflammatory effects of CR [22,23]. However, to date, whether CR inhibits neuroinflammation, neuronal loss and memory deficits induced by heat stress in animals remains unclear.

In this study, we aimed to investigate the effect of CR on (1) neuroinflammation, (2) neuronal death, and (3) memory dysfunction induced by heat stress in mice. To address underlying mechanisms in mice, the levels of HSP70, c-Fos, NF-κB, cortisol, tumor necrosis factor alpha (TNF-α), interleukin 1β (IL-1β), IL-9, IL-13, and prostaglandin E2 (PGE2) were measured in the blood and brains of mice exposed to heat. In addition, structural and behavioral changes in heat-stressed mice were examined using immunostaining and memory tests.

## 2. Materials and Methods

### 2.1. Animals

Animal maintenance and treatments were performed in accordance with the Animal Care and Use Guidelines of Kyung Hee University, Seoul, Korea (approved number; KHP-2014-05-3). Male imprinting control region (ICR) mice (seven weeks old, 30 g–32 g) were purchased from the Orient Co., Ltd., a branch of Charles River Laboratories (Seoul, Korea). The animals were housed at 10 individuals per cage (size 40 cm × 25 cm × 18 cm) with free access to water and food, and were kept under constant temperature (23 °C ± 1 °C) and humidity (60 ± 10%) and a 12-h light/dark cycle. After one week of arrival, the mice were adapted to their surroundings for seven days and kept under the same conditions before the start of the study.

### 2.2. Drugs

A dried rhizome of *Coptis chinensis* Franch was purchased from Omni Herb Inc. (Andong-si, Gyeongbuk, Korea). Air-dried products were cut into smaller pieces and pulverized to dry powder. The fibrous powder of the CR was extracted in boiling distilled water for 2 h. The lyophilized extract of CR was successively extracted with a yield of 11.42%. The reproducibility of CR was confirmed by producing a fingerprint using reference compounds: palmatine and berberine for CR, as described in our previous study [24]. The CR sample was deposited at the College of Pharmacy, Kyung Hee University.

### 2.3. Heat Exposure and Drug Administration

The mice were assigned to six groups (*n* = 10), treated as follows: Group 1 (control), Group 2 (vehicle), Group 3 (CR 30 mg/kg/day), Group 4 (CR 100 mg/kg/day), Group 5 (CR 300 mg/kg/day). The vehicle or CR of each sample dissolved in saline was administered by oral gavage for three days. Groups 2–5 were subjected to acute heat exposure (3 days) as previously described [5].

**Scheme 1:**
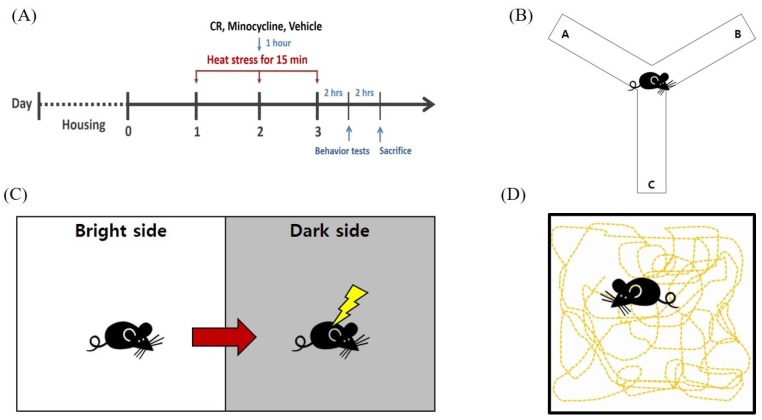
Diagrams of procedure and equipment in this study. (**A**) Scheme of the study, (**B**) Diagram of Y-maze task, (**C**) Diagram of passive avoidance test and (**D**) Diagram of open-field test.

Heat exposure was achieved by transferring the mice from their home cage into a chamber maintained at 43 °C and 60 ± 10% humidity for 15 min. To avoid the influence of diurnal cycling, heat exposure began at approximately the same time each day. Body temperature changes were measured using a TC-1000 temperature controller (CWE Inc., Ardmore, PA, USA) inserted into the ear and rectum after terminating heat stress. For the examination of protective effects of CR against astroglial activation and neuronal loss, male ICR mice were assigned to four groups, treated as follows: Group 1 (control), Group 2 (vehicle), Group 3 (minocycline 50 mg/kg/day; oral gavage), and Group 4 (CR 300 mg/kg/day). Groups 2, 3, and 4 were exposed to high temperature and treated with vehicle, minocycline or CR, respectively, once a day for three days (Scheme 1A).

### 2.4. Measurement of Cortisol Levels, TNF-α, IL-1β, IL-9, IL-13 and PGE_2_

A cortisol enzyme-linked immunosorbent assay (ELISA) assay (Enzo, New York, NY, USA) was performed according to the manufacturer’s protocol. Blood was collected from the mice (*n* = 10) on the day of decapitation, and centrifuged at 3000 rpm for 10 min to obtain serum samples, which were stored at −70 °C until use. Briefly, serum was mixed with diethyl ether. Then, ether mixture was evaporated using nitrogen, after which protease activity was detected using a microplate reader (VERS Amax, Sunnyvale , CA, USA), with filters set at 570 nm excitation and 590 nm emission.

The mouse TNF-α, IL-1β (Ray Biotech, Norcross, GA, USA), IL-9, IL-13, and PGE_2_ ELISA kits (Enzo, New York, NY, USA) were performed according to the manufacturer’s protocol. Briefly, the hypothalamic or hippocampal lysates were incubated with reaction buffer. The mixture was incubated for 2.5 h at room temperature before protease activity was detected using a microplate reader, with filters set at 360 nm excitation and 450 nm emission. The mixture was incubated for 45 min at room temperature before protease activity was detected using a microplate reader, with filters set at 570 nm.

### 2.5. Western Blotting

Western blotting was performed according to the method described previously [25]. Brain tissues were lysed using a protein assay kit according to the manufacturer’s instructions. The lysates were separated by 10% sodium dodecyl sulfate-polyacrylamide gel electrophoresis (SDS-PAGE) and then transferred to a polyvinylidene difluoride membrane (Millipore Bioscience Research). The membranes were incubated with 5% skim milk in Tris-buffered saline with Tween 20 (TBST) for 1 h and then with a primary antibody (1:1000; HSP70, c-Fos or NF-κB, and iNOS, Santa Cruz, Pas Robles, CA, USA) overnight at 4 °C; this was followed by incubation with a horseradish peroxidase conjugated secondary antibody for 1 h. Immunoreactive-bands were detected using an enhanced chemiluminescence (ECL) detection kit (Bionote, Hwaseong, Korea), and a LAS-4000 Mini system (Fujifilm Corp., Tokyo, Japan) was used for visualization. Band intensities were normalized to the β-actin band intensity using MultiGauge software (Fujifilm Corp.).

### 2.6. Behavior Tests

#### 2.6.1. Passive Avoidance Test

The cognitive function was assessed using a two-compartment step-through passive avoidance apparatus. The box was divided by a guillotine door into bright and dark compartments (21 cm × 21 cm × 21 cm). The bright compartment contained an electric lamp, and the floor of the dark compartment was composed of 2-mm stainless steel rods spaced 1 cm apart. The door between the two compartments was opened 10 s later. Then, when the hind legs of the mice entered the dark chamber, the guillotine door was closed, and an electrical foot shock (0.6 mA) was delivered through the grid floor for 3 s (Scheme 1C). The mice were again placed in the bright chamber for the retention trial, 24 h after the acquisition trial. The time taken for a mouse to enter the dark chamber after the door opening was defined as the latency time, which was recorded for up to 300 s.

#### 2.6.2. Y Maze Task

To evaluate short-term spatial memory function, we performed the Y-maze task. The Y-maze task apparatus was composed of a three arms with equal angles from each other (40 cm long and 3 cm wide with 12 cm high walls) as described previously [26]. Each arm had a sequence such as A, B, and C, and mice were placed within any arms (Scheme 1B). Numbers of arm entries were recorded manually for each mouse over 8 min periods. The actual alternation was defined as entries into all three arms on consecutive choices, such as ABC, CAB, or BCA. The result was expressed as the percentage of alternation identified by the following equation: % Alternation = ((Number of alternations)/(Total arm entries − 2)) × 100.

#### 2.6.3. Open-Field Test

The open-field test is a classic method to measure the ambulation of mice. For excluding behavior results related to sickness, we performed the open-field test. The mice were placed in a testing chamber (40 cm × 25 cm × 18 cm) with a black floor for 5 min adaptation, followed by a 30 min test period using a computerized automatic analysis system (Viewer; Biobserve, Bonn, Germany). The data collected by computer included the total distance traveled by tracking the center of the animal (Scheme 1D).

### 2.7. Immunohistochemistry and Immunofluorescence

Two hours after the behavioral test, mice were anesthetized by Avertin (12.5 mg/kg) and perfused transcardially with 0.05 M phosphate buffered saline (PBS), and then fixed with cold 4% paraformaldehyde (PFA) in 0.1 M phosphate buffer. Brains were removed and post-fixed in 4% PFA overnight at 4 °C, and then immersed in a solution containing 30% sucrose in 0.05 M PBS for cryoprotection. Serial 30 μm-thick coronal sections were cut on a freezing microtome (Leica, Nussloch, Germany) and stored in cryoprotectant (25% ethylene glycol, 25% glycerol, and 0.05 M phosphate buffer) at 4 °C until use. For immunohistochemical study, brain sections were treated with 1% hydrogen peroxide for 15 min. The sections were incubated with a primary antibody (NeuN, synaptophysin and post-synaptic density (PSD-95), 1:500, Millipore, Billerica, MA, USA) overnight at 4 °C, in the presence of 0.3% Triton X-100 and normal goat serum. The sections were then incubated with biotinylated anti-mouse IgG (1:200 dilution) for 90 min, and with avidin-biotin complex (1:100 dilution, Vector Labs, Burlingame, CA, USA) for 1 h at room temperature. Peroxidase activity was visualized by incubating sections with 3,3-diaminobenzidine in 0.05 M Tris-buffered saline (pH 7.6). After several rinses with PBS, sections were mounted on gelatin-coated slices, dehydrated, and slipped with a cover slide using a histomount medium.

For immunofluorescence study, brain sections were incubated overnight at 4 °C with anti-glial fibrillary acidic protein (GFAP) antibody (dilution 1:500, Millipore, MA, USA). They were then incubated for 2 h with an Alexa Fluor 594-conjugated horse anti-goat IgG (dilution 1:500). The sections were finally washed in PBS and mounted using Vectashield Mounting Medium. Confocal immunofluorescent images were captured using an LSM 700 confocal microscope (Carl Zeiss, Thornwood, NY, USA). Data are presented as percentages of control values.

To measure immunoreactivity, the total region of interest was outlined manually, and the averaged optical densities and cell counts were measured using ImageJ software (Bethesda, MD, USA).

### 2.8. Statistical Analysis

All statistical parameters were calculated using GraphPad Prism 5.0 software (Graphpad Software, San Diego, CA, USA). All quantifications for assays were repeated three times and values were expressed as the mean ± SEM. All data were tested for normal distribution using Shapiro-Wilk test. All of data from Western blot analyses, ELISA, behavior tests, immunohistochemistry and immunofluorescence analyses were analyzed by one-way analysis of variance followed by Tukey’s post hoc test. Differences with a *p* value less than 0.05 were considered statistically significant.

## 3. Results

### 3.1. Inhibitory Effects of CR on Increased Body Temperature Induced by Heat Exposure

**Figure 1:**
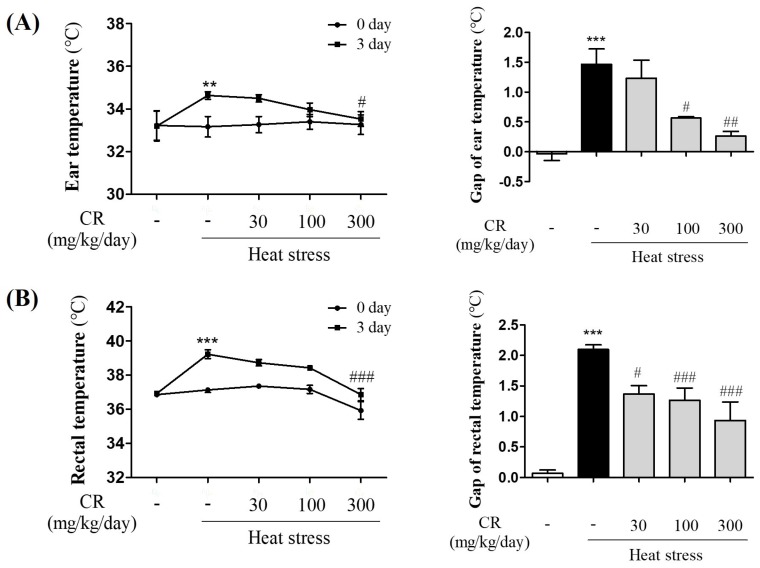
Inhibitory effects of Coptidis Rhizoma (CR) on heat exposure-induced hyperthermia. The white, black and gray bars indicate the control group, the heat-stressed vehicle-treated group and heat-stressed CR-treated groups, respectively. (**A**) Treatment with CR significantly lowered the heat exposure-induced elevation of ear temperature in a dose-dependent manner. (**B**) The temperatures of the CR-treated group were significantly lower than that of the heat-stressed vehicle-treated group in dose-dependent manner. Values are expressed as means ± SEM. ** *p* < 0.01 and *** *p* < 0.001 as compared with the control group. ^#^ *p* < 0.05, ^##^ *p* < 0.01 and ^###^ *p* < 0.001 as compared with the heat-stressed vehicle-treated group.

Numerous studies have shown that heat stress induces many physiological modifications, including water loss, anorexia, weight loss, increased heart rate, and hypoglycemia [1,2,27,28,29,30]. Additionally, CR reportedly can reduce fever temperature, and has been for the treatment of heat disorders [19]. To evaluate the antipyretic effects of CR on heat stress-induced hyperthermia, we assessed ear and rectal temperatures. The ear temperature was significantly elevated in the heat-stressed vehicle-treated group (1.47 °C; *n* = 10) compared with the control group (−0.03 °C; *n* = 10), whereas ear temperature decreased in the CR-treated groups dose-dependently at 30, 100 and 300 mg/kg/day (1.23, 0.57, and 0.27 °C, respectively; *n* = 10; Figure 1A). Furthermore, the rectal temperature significantly increased in the heat-stressed vehicle-treated group (2.1 °C) compared with the control group (0.07 °C). Conversely, rectal temperature decreased in CR-treated groups dose-dependently at 30, 100, and 300 mg/kg/day (1.37, 1.27, and 0.93, respectively, Figure 1B). These physiological responses of vehicle-treated and CR-treated groups under our experimental heat stress indicated that (1) our experimental conditions (43 °C and 60 ± 10% humidity for 15 min) were appropriate to induce hyperthermia, and (2) CR exhibited inhibitory effects on elevated body temperature induced by heat exposure, suggesting that CR can act as a deterring agent against heat stress.

### 3.2. Inhibitory Effects of CR on Heat Stress-Induced Secretion of Cortisol

**Figure 2:**
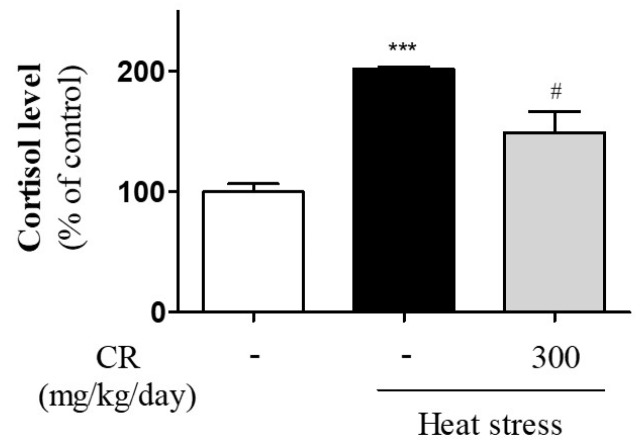
Repressive effect of CR on heat exposure-induced enhancement of cortisol secretion. The white, black and gray bars indicate the control group, the heat-stressed vehicle-treated group and heat-stressed CR-treated groups, respectively. Values are expressed as means ± SEM. *** *p* < 0.001 as compared with the control group. ^#^ *p* < 0.05 as compared with the heat-stressed vehicle-treated group.

Cortisol is a glucocorticoid stress hormone secreted from the cortex of the adrenal gland. Heat stress induces increased cortisol levels [31,32], which can be used as an index of heat stress. To evaluate the effects of CR on hyperthermia-induced release of cortisol, we examined cortisol levels in mouse serum. The cortisol secretion in blood was significantly increased in the heat-stressed vehicle-treated group (201.92%) compared with the control group (100%). However, the cortisol levels were significantly decreased in the CR-treated groups at 300 mg/kg/day (149.19%; Figure 2). These results indicate that CR inhibits elevated cortisol secretion induced by heat stress.

### 3.3. Inhibitory Effects of CR on Heat Stress-Induced Biochemical Changes in the Brain

**Figure 3:**
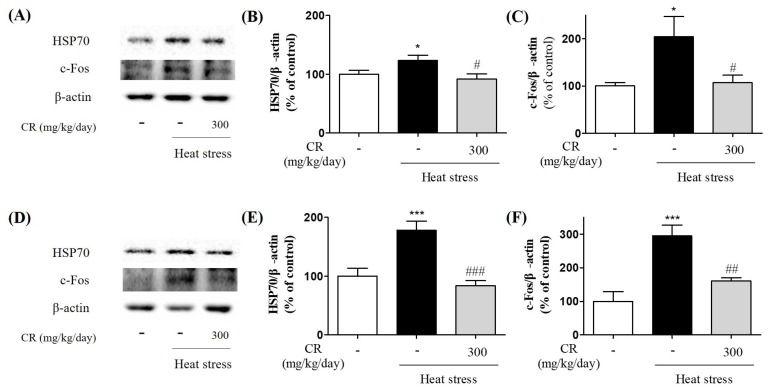
Inhibitory effects of CR against thermal stress-induced up-regulation of heat shock protein and activation of immediate early gene in the hypothalamus and hippocampus of mice. (**A**,**D**) Both HSP70 and c-Fos expressions were assessed using Western blotting with antibodies against HSP70 and c-Fos in hypothalamus and hippocampus, respectively. Quantification of densitometric analyses was performed by measuring the expression ratios of HSP70/β-actin and c-fos/β-actin in the lysates of hypothalamus (**B**,**C**) and hippocampus (**E**,**F**). Values are expressed as means ± SEM. * *p* < 0.05 and *** *p* < 0.001 as compared with the control group. ^#^ *p* < 0.05, ^##^ *p* < 0.01 and ^###^ *p* < 0.001 as compared with the heat-exposed vehicle-treated group.

Many reports have demonstrated that heat stress causes the upregulation of HSP70 and c-Fos expression in the hypothalamus [5,33]. To evaluate the effects of antifebrile CR on heat stress-related biological responses in the brain, we performed Western blotting to measure the HSP70 and c-Fos expression in the hypothalamus and hippocampus. Western blot analysis showed that heat-exposed vehicle-treated group exhibited a trend towards upregulation of HSP70 and c-Fos in the mouse hypothalamus and hippocampus (Figure 3). Interestingly, CR-treated groups showed a significant reduction of HSP70 and c-Fos expression compared with the heat-exposed vehicle-treated group (Figure 3). HSP70 expression levels were the highest in the vehicle-treated group and reduced following CR treatment at 300 mg/kg/day (Figure 3B,E). The c-Fos levels showed a similar tendency (Figure 3C,F). These findings indicate that CR inhibits heat stress-related biological responses in the brain, suggesting that CR may work as a relieving factor of heat stress.

### 3.4. Attenuating Effects of CR on Heat Stress-Induced Production of Inflammatory Mediators

**Figure 4:**
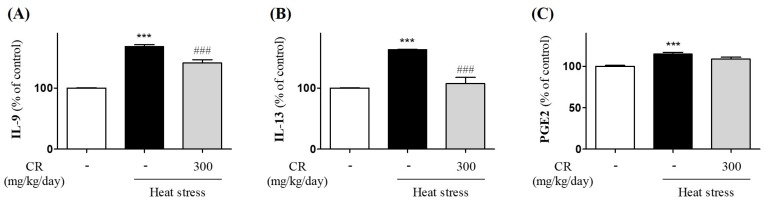
Effects of CR on the elevated release of IL-9, IL-13, and PEG2 in hypothalamus of mice after heat exposure. In hypothalamus of mice after heat exposure, the presence of cytokines was determined using sandwich ELISA kit. (**A**) The release of IL-9 was significantly increased after heat exposure, however, but 300 mg/kg of CR significantly reduced the heat stress-induced elevation of IL-9. (**B**) The production of IL-13 was elevated under heat-stress conditions, whereas the treatment of CR significantly decreased the elevated level of IL-13. (**C**) In the hypothalamus of mice, the presence of PGE2 was determined using sandwich ELISA analysis. Values are expressed as means ± SEM. *** *p* < 0.001 as compared with the control group. ^###^ *p* < 0.001 as compared with the heat-exposed vehicle-treated group.

Next, to determine the effects of CR on heat stress-induced inflammatory cytokines in the hypothalamus, we examined IL-9 and IL-13 expression levels using ELISA analysis. Both expression levels were significantly increased in the hypothalamic homogenates of mice subjected to heat stress (Figure 4A,B). However, the IL-9 expression was significantly decreased in the CR-treated groups at 300 mg/kg/day (Figure 4A). Moreover, IL-13 expression was significantly increased in the heat-stressed vehicle-treated group, and the IL-13 levels significantly decreased in the CR-treated groups at 300 mg/kg/day, showing a dose-dependent tendency (Figure 4B). These findings indicate that CR has inhibitory effects on heat stress-induced production of inflammatory cytokines in the hypothalamus. PGE2 has direct roles in the genesis of fever [34,35]. Additionally, PGE2 is abundantly produced in the brain in response to inflammation and mimics stress responses [36]. To evaluate the effects of antipyretic CR on heat stress-induced PGE2 production in the hypothalamus, we examined PGE_2_ expression using ELISA analysis. PGE2 expression was significantly upregulated after heat exposure and vehicle treatment but PGE2 upregulation was inhibited in the CR-treated groups (Figure 4C). These data indicate that CR has inhibiting activities against inflammation-related fever genesis.

### 3.5. Suppressive Effects of CR on Heat Stress-Induced Gliosis in Hippocampus

**Figure 5:**
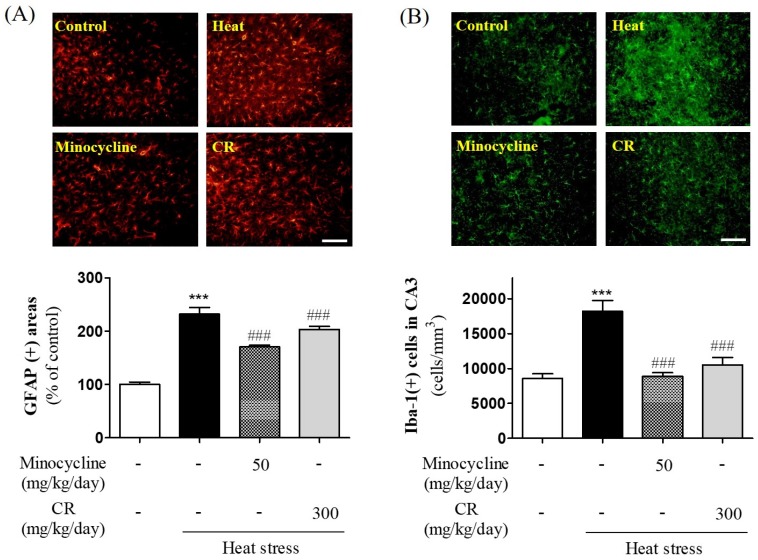
Inhibitory effects of CR on heat stress-induced neuroinflammation in hippocampus of mice. (**A**) The effect of CR (300 mg/kg) on astrogliosis was determined using GFAP immunostaining. (**B**) The effect of CR (300 mg/kg) on microgliosis was determined using Iba-1 immunostaining. The minocycline (50 mg/kg), an anti-inflammatory drug, was used as positive control. Scale bar = 50 μm. Values are expressed as means ± SEM. *** *p* < 0.001 as compared with the control group. ^###^ *p* < 0.001 as compared with the heat-stressed vehicle-treated group.

In our recent study, we demonstrated that heat stress potently induces neuroinflammation in the hippocampus, and hyperthermia-mediated glial activation can act as a causative factor for neuronal death [5]. Thus, to investigate the effects of antipyretic CR on heat stress-induced astrogliosis and microgliosis in the hippocampal CA3 region, we performed immunofluorescence and immunoperoxidase analyses (Figure 5). First, we investigated the effects of CR on heat stress-induced astroglial activation in the hippocampus using immunohistochemistry with an antibody against GFAP. Immunohistochemical analysis revealed that the number of GFAP-stained astrocytes was increased in the hippocampus following heat exposure (Figure 5A). However, CR administration (300 mg/kg) significantly attenuated astrocyte activation triggered by heat stress (Figure 5A). Additionally, the positive control minocycline (50 mg/kg), an anti-inflammatory agent, also repressed heat stress-induced astrogliosis in the hippocampus. Next, we investigated the effects of CR on heat stress-induced microglial activation in the hippocampus using immunohistochemistry with an antibody against Iba-1. Immunohistochemical analysis revealed the number of Iba-1-stained microglial cells was increased in the hippocampus following heat exposure (Figure 5B). However, CR administration (300 mg/kg) significantly attenuated the astrocyte activation triggered by heat stress (Figure 5B). For the first time, we demonstrated that CR might lead to suppression of glial activation induced by heat stress in the hippocampus.

### 3.6. Inhibiting Effects of CR on Heat Stress-Induced NF-κB Activation and Production of Inflammatory Mediators

**Figure 6:**
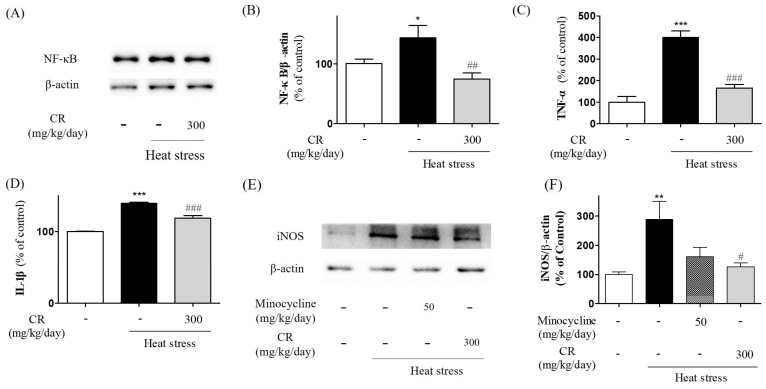
Effects of CR on thermal stress-induced NF-κB activation and up-regulation of pro-inflammatory mediators. (**A**) Representative Western blot analysis of the expression of NF-κB in the hippocampus. (**B**) Quantification of densitometric analyses was performed by measuring the expression ratios of NF-κB to β-actin. (**C**,**D**) The expression of TNF-α and IL-1β were assessed by sandwich ELISA. (**E**) Representative Western blot analysis of the expression of iNOS in the hippocampus. (**F**) Quantification of densitometric analyses was conducted by measuring the expression ratios of iNOS to β-actin. Values are expressed as means ± SEM. * *p* < 0.05, ** *p* < 0.01 and *** *p* < 0.001 as compared with the control group. ^#^ *p* < 0.05, ^##^ *p* < 0.01 and ^###^ *p* < 0.001 as compared with the heat-exposed vehicle-treated group.

NF-κB is an important upstream modulator of proinflammatory molecules such as TNF-α and IL-1β [37]. Moreover, NF-κB can be activated by heat exposure in the hippocampus [5] and influence synaptic plasticity and memory [38]. To determine the effects of CR on heat stress-induced NF-κB activation in the hippocampus, we examined NF-κB expression using Western blot analysis (Figure 6A,B). The NF-κB/β-actin ratios were increased under heat exposure, while CR treatment significantly decreased at doses of 300 mg/kg/day, compared with the heat-exposed vehicle-treated group (Figure 6B). These data indicated that CR can downregulate the NF-κB pathway, contributing to inhibition of proinflammatory molecule production in the brain.

Heat stress induces the production of inflammatory mediators such as inducible nitric oxide synthase (iNOS), cyclooxygenase 2 (COX-2) and cytokines [5]. To evaluate the effects of antifebrile CR on heat stress-mediated release of inflammatory cytokines in the hippocampus, we measured the levels of cytokines such as TNF-α and IL-1β using ELISA. The TNF-α levels were significantly increased in the heat-exposed group compared with the control group, and significantly decreased in the heat-exposed CR-treated groups at 300 mg/kg/day (Figure 6C). Additionally, IL-1β levels were significantly enhanced after heat exposure, but significantly decreased in the heat-exposed CR-treated group at 300 mg/kg/day (Figure 6D). To investigate whether CR could inhibit up-regulation of iNOS induced by thermal stress, we detected the expression of iNOS in hippocampal region by Western blot. The expression of iNOS was significantly increased in the heat-exposed group compared to the control group. In contrast, CR-treated group at 300 mg/kg/day showed the significant decrease of iNOS expression compared with heat-exposed group as well as the positive control-treated group (minocycline at 50 mg/kg/day; Figure 6E,F). These findings suggest that CR has inhibitory effects on heat stress-induced production of inflammatory molecules in the hippocampus.

### 3.7. Atternuating Effects of CR on Heat Stress-Induced Neurodegeneration in Hippocampus

**Figure 7:**
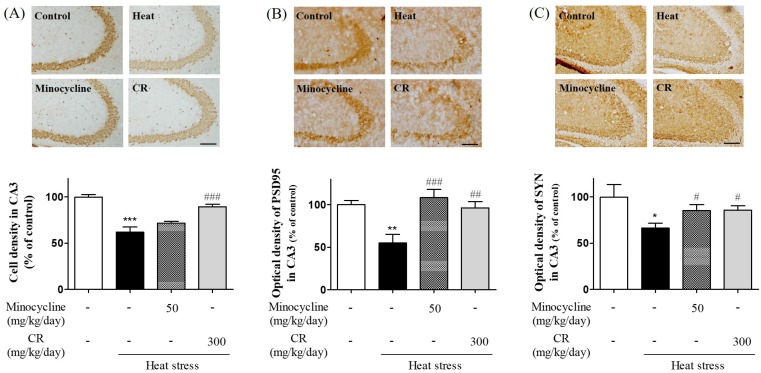
Inhibitory effects of CR on heat stress-induced neurodegeneration in hippocampus of mice. (**A**) The effect of CR (300 mg/kg) on neuronal loss was determined using NeuN immunostaining. (**B**,**C**) The effect of CR (300 mg/kg) on synaptic function was measured with PSD-95 and synaptophysin immunostaining. (**B**) Representative pictures of PSD-95 staining are shown. (**C**) Representative pictures of synaptophysin staining are shown. Scale bar = 50 μm. The minocycline (50 mg/kg), an anti-inflammatory drug, was used as positive control. Values are expressed as means ± SEM. * *p* < 0.05, ** *p* < 0.01 and *** *p* < 0.001 as compared with the control group. ^#^ *p* < 0.05, ^##^ *p* < 0.01 and ^###^ *p* < 0.001 as compared with the heat-stressed vehicle-treated group.

To examine the effects of CR on neuronal and synaptic loss, brain sections were stained with the neuronal marker NeuN and synaptic markers, such and PSD-95 and synaptophysin. Under heat-stressed conditions, NeuN-stained neuronal cells were significantly reduced in the hippocampal CA3 region compared with control animals. Interestingly, CR at 300 mg/kg/day significantly increased neuronal density in the hippocampus (Figure 7A). Additionally, minocycline at 50 mg/kg reduced the density of NeuN-positive cells compared with the heat-stressed vehicle treated group (Figure 7A). Neuroinflammation induces the alternation and reduction of synaptic markers, such as synaptophysin and PSD-95, resulting in cognitive deficits [39]. To evaluate the effect of CR on the reversal of synaptic molecules, we detected the optical density of synaptophysin and PSD-95 in hippocampal CA3 region compared with control animals. Both synaptic proteins were significantly recovered in CR at 300 mg/kg/day group, which were decreased in heat-exposed group (Figure 7B,C). Minocycline at 50 mg/kg/day group showed the similar tendency with CR-treated group for attenuating the reduction of synaptophysin and PSD-95 (Figure 7B,C). For the first time, we demonstrated that CR might lead to protection against neuronal and synaptic loss induced by heat stress in the hippocampus.

### 3.8. Inhibitory Effects of CR against Hyperthermia-Mediated Memory Impairment

**Figure 8:**
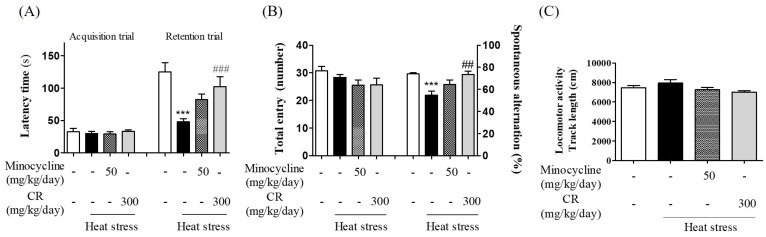
Protective effects of CR on thermal stress-induced cognitive deficits. (**A**) We determined cognitive function using a passive avoidance task. (**B**) We also performed the Y-maze task to evaluate the memory function. (**C**) We conducted the open-field test to rule out the heat-induced sickness behavior. Values are expressed as means ± SEM. *** *p* < 0.001 as compared with the control group. *^##^ p <* 0.01 and ^###^ *p* < 0.001 as compared with the heat-exposed vehicle-treated group.

Heat stress and neuroinflammation cause cognitive dysfunction [5,40]. Therefore, to investigate whether the antipyretic and anti-inflammatory CR can protect against heat stress-induced memory impairment, we conducted a passive avoidance test to assess learning ability and memory in mice. During the acquisition trial, no significant differences between the groups were detected (Figure 8A). However, the retention time of the heat-exposed vehicle treated group was significantly decreased. Moreover, the retention time of the heat-exposed CR-treated group at 300 mg/kg/day was significantly longer than the heat-exposed vehicle-treated group, showing a more potent effect than minocycline (Figure 8A). The retention time of the heat-exposed minocycline-treated group at 50 mg/kg/day was longer than the heat-exposed vehicle-treated group. To confirm the effect of CR on cognitive behavior, the Y maze test was also performed. The percentage of spontaneous alternation was significantly decreased in heat-exposed vehicle-treated group. However, the heat-exposed CR-treated group at 300 mg/kg/day showed significant amelioration of memory deficits, compared with the heat-exposed vehicle-treated group (Figure 8B). The percentage of spontaneous alternation of the heat-exposed minocycline-treated group was higher than that of the heat-exposed vehicle treated group. The total entries of each group had no differences. Finally, we conducted the open-field test to examine the influence of both heat-exposure and CR administration on locomotor activity. As the results of locomotor activity, there was no difference between the control group and heat-exposed vehicle-treated group (Figure 8C). In addition, the mice of heat-exposed minocycline- or CR-treated groups had similar movements with those of control group. These findings indicate that antipyretic CR not only inhibits neuroinflammation and neurodegeneration induced by heat stress but also attenuates hyperthermia-triggered cognitive deficits.

## 4. Discussion

**Figure 9:**
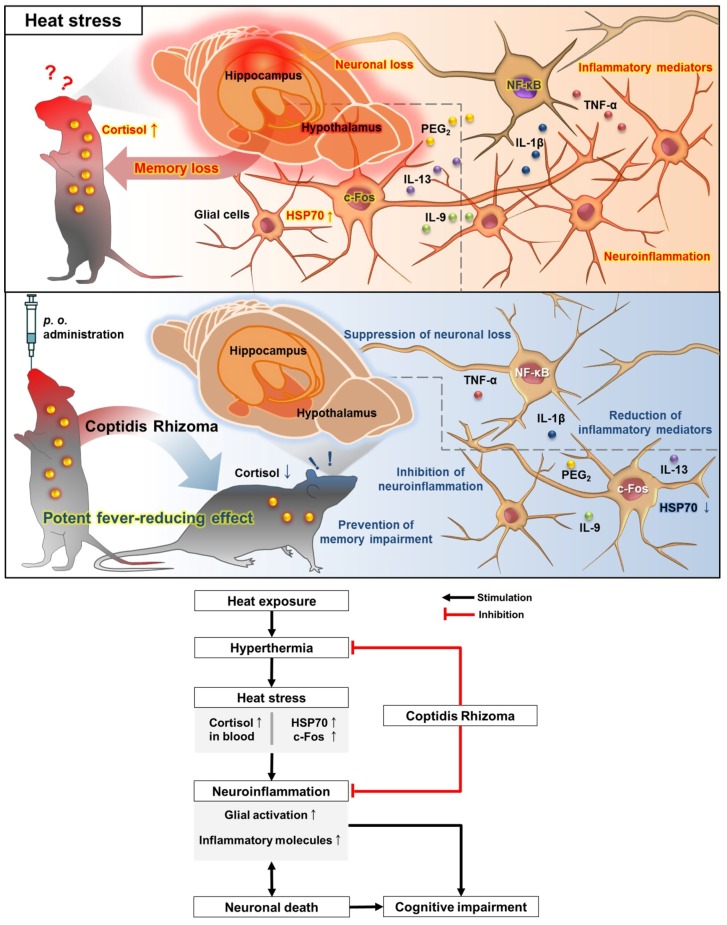
Schematic diagram of the protective effects and proposed therapeutic mechanisms of Coptidis Rhizoma against heat stress-induced damages in the brain of mice.

In the present study, we investigated whether CR exerts protective effects against brain damage resulting from heat-stress via antipyretic effects in mice. This study clearly showed that CR has potent antifebrile effects on elevated body temperature, and inhibitory actions on stress responses and hormonal and biochemical changes after heat exposure. To reveal the anti-inflammatory effects of CR, we observed the inhibition of astrogliosis and reduced NF-κB, TNF-α, IL-1β, IL-9, IL-13 and PGE_2_ levels, resulted in neuronal death and memory decline induced by heat stress in mice. Therefore, the cognitive improvement after treatment with CR appears mediated by its antipyretic activities (Figure 9).

In our recent study, heat exposure strongly induced stress responses, resulting in increased stress-related physiological factors such as cortisol in blood and HSP70 and c-Fos in the hypothalamus of mice [5]. Additionally, heat stress can cause glial activation with NF-κB pathway in the hippocampus, which subsequently led to the upregulation of TNF-α, IL-1β, iNOS and COX-2, resulting in neurodegeneration and cognitive impairment. These findings suggest that heat exposure might damage cognitive functions by inducing stress responses, neuroinflammation and neurodegeneration in mouse brains.

Heat exposure induces stress-related physiological, hormonal, and biochemical changes in rodents [41]. Additionally, the stress responses are significantly involved in the complex HPA axis [42]. The HPA axis is a key player in responses to stressful stimuli and includes the autonomic nervous system, which can self-activate through neurotransmitters [43]. Glucocorticoids are hormones controlled by the HPA axis and secreted in response to stress [44]. Cortisol, another steroid hormone, is an important factor of glucocorticoid hormone synthesis in the brain [45]. The major physiological role of cortisol is the regulation of homeostasis and stress response in the body [46]. Furthermore, when cortisol binds to the cytoplasmic glucocorticoid receptor (GR)/heat shock protein (HSP) complex, dissociation of the HSP complex occurs [47]. Subsequently, the GR-cortisol complex facilitates the repression of proinflammatory transcription factors such as c-Fos [48]. In the present study, oral administration of CR significantly inhibited the heat stress-induced increase of body and rectal temperatures. Results coincided in previous reports that cortisol secretion, and HSP70 and c-Fos expressions were shown to be triggered by heat exposure in the hypothalamus of mice [5]. However, treatment with CR significantly reduced the release of cortisol and upregulation of HSP70 and c-Fos in the present study (Figure 2 and Figure 3). Based on these results, CR may play an important role in the HPA axis impaired by heat stress.

Glial cells play an important role in inflammatory reactions in the central nervous system [49]. Inflamed glial cells produce diverse inflammatory mediators such as NO, TNF-α, IL-1β and PGE_2_, resulting in NF-κB-associated neurotoxicity followed by activation of the HPA axis [24]. Numerous studies have shown that heat stress increases the expressions of NF-κB, TNF-α and IL-1β from activated glial cells and the release of proinflammatory molecules, such as IL-9, IL-13 and PGE_2_ [5,50,51]. Additionally, various studies have shown anti-inflammatory activities of CR in humans and animals [23]. In this study, heat exposure increased the levels of TNF-α and IL-1β expression and those mediators were significantly reduced by treatment with CR (Figure 6). Furthermore, treatment with CR significantly suppressed the proinflammatory responses in the hypothalamus of mice (Figure 4). Collectively, these results indicate that treatment with CR inhibits inflammatory mediators induced by heat stress in mouse brains.

Numerous studies have shown that activated glial cells in the brain produce neurotoxic factors that subsequently lead to neuronal death [52,53,54]. Additionally, in the hippocampus, heat stress leads to activated glial cells and increased inflammatory molecules, causative factors of memory loss and neuronal death [5]. To examine the anti-inflammatory and memory-enhancing effects of CR, minocycline was chosen as a positive control. Minocycline, a broad-spectrum tetracycline antibiotic, is widely used as a positive control for screening of inflammation and cognitive activities [55]. Minocycline significantly reduced the number of activated microglia and astrocytes in Aβ_1-42_-injected rat brains [56]. Moreover, minocycline may exert neuroprotective effects against anti-inflammatory responses [57]. To date, whether CR administration can reduce heat stress-mediated astrocyte activation in the mouse brain remains unclear. In the present study, treatment with minocycline and CR inhibited glial activation and memory loss induced by heat exposure in mice (Figure 5 and Figure 8).

Traditionally, CR is one of the most frequently used medicinal plants, and numerous studies have reported various pharmacological activities of CR [58]. Since CR has various functions besides its antifebrile qualities, it may affect heat stress-mediated brain pathology through other properties. For example, CR showed inhibitory effects on the NF-κB pathways in vitro and the expression of proinflammatory molecules in vivo [59,60]. Moreover, several bioactive constituents in CR have anti-inflammatory, antibacterial, and antioxidant effects [59,61]. Clinically, CR has therapeutic effects on hypertension and Alzheimer’s disease [62,63].

Reportedly, heat exposure might be associated with deficits in short-term memory [64]. Additionally, inflammatory responses can directly contribute to cognitive impairment and neuronal death in animals [5]. In the present study, CR exhibited inhibitory effects on heat stress-induced cognitive deficits and neurodegeneration in the hippocampus of mice (Figure 7 and Figure 8). Previous studies demonstrated that CR has anti-inflammatory effects by suppressing the NF-κB pathway and inhibiting proinflammatory protein expressions [59,60]. Additionally, CR exhibits anti-inflammatory effects by suppressing the NF-κB pathway and inhibiting the proinflammatory protein expression induced by heat stress. These findings indicate that the antipyretic and anti-inflammatory CR is capable of attenuating hyperthermia and inflammation-mediated memory loss. However, further studies are needed to elucidate the direct effects of CR on cognitive functions and their specific mechanisms.

In the present study, CR exhibited anti-inflammatory effects in the brain with heat stress-induced damages. Since neuroinflammation or activated glial cells can contribute to reduction in adult neurogenesis, anti-inflammatory effect of CR is, apparently, a positive stimulator of heat stress-induced impairment of adult neurogenesis. Thus, further studies are necessary to clarify the direct relationship between CR and adult neurogenesis. Reportedly, enhancement of adult hippocampal neurogenesis can promote learning and memory, and further studies may elucidate whether CR can improve cognitive function through enhanced adult neurogenesis.

The development of nootropic agents has attracted much attention from many researchers for the treatment of neurodegenerative diseases. Many researchers have evaluated the relationship between CR and the treatment of dementia focusing on alkaloids, the component of CR associated with neuroprotective properties [61,65,66,67,68,69]. Although the heat-lowering effect appears irrelevant to dementia, for the first time, this study demonstrated the memory-enhancing effect of CR in animals. Furthermore, the rhizome of *Coptis chinensis* can reduce stress-induced neuroinflammation and cell death. These anti-inflammatory and neuroprotective roles could result in using CR as a new drug candidate for neurodegenerative diseases.

In summary, the rhizome of *Coptis chinensis* exhibits attenuating effects on heat stress-induced physiological, hormonal and biochemical responses inhibiting the production of inflammatory mediators through deactivation of glial cells. Moreover, the rhizome of *Coptis chinensis* protected against neuronal damage and cognitive dysfunction induced by heat stress in mice (Figure 9). These results suggest that CR could be a promising therapeutic candidate for heat-related memory disorders and/or inflammatory diseases.

## Abbreviations

CR	Coptidis Rhizoma
IL	Interleukin
HPA	Hypothalamic-pituitary-adrenal
HSP70	Heat shock protein 70
NF-κB	nuclear factor kappa β
TNF-α	tumor necrosis factor alpha
PGE_2_	prostaglandin E_2_
iNOS	inducible nitric oxide synthase
COX-2	cyclooxygenase 2
GR	glucocorticoid receptor
ELISA	Enzyme-linked immunosorbent assay
GFAP	Glial fibrillary acidic protein
